# Stable two- and three-dimensional cholangiocyte culture systems from extrahepatic bile ducts of biliary atresia patients: use of structural and functional bile duct epithelium models for in vitro analyses

**DOI:** 10.1007/s10616-024-00620-7

**Published:** 2024-04-08

**Authors:** Ai Shimamura, Mayumi Higashi, Kazuya Nagayabu, Shigeru Ono

**Affiliations:** 1 Department of Pediatric Surgery, Kyoto Prefectural University of Medicine, Kyoto, Japan; 2 Department of Emergency and Critical Care Medicine, Hiroshima University, 1-2-3 Kasumi, Minami-ku, Hiroshima, 734-8551 Japan

**Keywords:** Biliary atresia, Cholangiocyte, Cell culture, Three-dimensional

## Abstract

We herein report two- (2D) and three-dimensional (3D) culture methods of cholangiocytes originating from extrahepatic bile ducts of biliary atresia (BA) patients. Cells were stabilized for in vitro analyses, and 3D culture by two different methods showed the structural and functional features of cholangiocytes in the gel scaffold. First, cells were obtained from gallbladder contents or resected tissues of patients at surgery and then cultured in our original conditioned medium with a cocktail of signaling inhibitors that maintains the immaturity and amplification of cells. Cells were immortalized by inducing SV40T and hTERT genes using lentivirus systems. Immunostaining with CK19 and Sox9 antibodies confirmed the cells as cholangiocytes. 3D organoids were formed in Matrigel in two different ways: by forming spheroids or via vertical growth from 2D cell sheets (2 + 1D culture). Organoids generated with both methods showed the uptake and excretion of rhodamine-123, and duct-like structures were also found. Our culture methods are simpler than previously reported methods and still show the structural and functional characteristics of cholangiocytes. Thus, this system is expected to be useful for the in vitro investigation of cholangiocyte damage or regeneration in BA patients.

## Introduction

Biliary atresia (BA) is a cholestatic disease caused by obstruction of extrahepatic bile ducts in early infants. The bile tracts have usually been destroyed by the time of the diagnosis and replaced with fibrotic tissues (Asai et al. 2015; Ortiz-Perez et al. 2020; Gunadi et al. 2020). Patients develop cholestatic liver failure and suffer a fatal outcome if surgical intervention is not performed within the first few months of life.

The primary surgical approach for managing BA patients is the Kasai procedure (hepatic portoenterostomy), in which the fibrotic remnants of the bile ducts at the portal area of the patient’s liver are resected, and the jejunum is attached as an excretion tract for bile (Ohi 2001; de Carvalho et al. 2019). However, even after the Kasai procedure, bile excretion from the liver is often insufficient to prevent cirrhosis development over time, and approximately 60% of patients need liver transplantation at some point in their life (Kasahara et al. 2017; Rodijk et al. 2020; Ziogas et al. 2021).

Although the etiology has long been studied from various perspectives (e.g. genetic, infectious, immunological, etc.), the cause of bile duct damage has not yet been clarified (Bezerra et al. 2018; Lakshminarayanan et al. 2016). From a pathological perspective, the epithelia of damaged extrahepatic bile ducts are mostly peeled off in the lumen and replaced with fibroblasts in BA patients. Inflammatory cells, including neutrophils, eosinophils, and mast cells, aggregate around the damaged bile ducts and blood vessels, which show evidence of inflammatory reactions at the site of obstruction (Asai et al. 2015; Ortiz-Perez et al. 2020). However, these findings do not indicate the specific cause of bile duct damage in BA patients.

One issue encountered when studying BA is the lack of materials. Resected remains of bile ducts or tissues of livers are damaged at the time of surgery and do not reveal the process of the damage to the bile duct epithelium. Studying the dynamics of living cells in vitro might reveal the details of the onset or progression of damage in the epithelial cells of bile ducts. Furthermore, studying the growth of cholangiocytes would indicate its effectors, such as accelerators or inhibitors of regeneration after surgery. However, even normal cholangiocytes are difficult to obtain as cultured cells for in vitro experiments, much less specific cells from BA patients.

While researching the process of extrahepatic bile duct damage, we managed to establish stabilized cell culture methods for cholangiocytes from the extrahepatic bile ducts of BA patients and utilized these cells for BA research in two-dimensional (2D) and three-dimensional (3D) ways. Primary cells were obtained at the time of surgery without the need for any complicated procedures and grown relatively easily with clusters of immature cells in our conditioned medium. These cells are useful for in vitro experiments, such as examining reactions to cytokines or chemicals, or for conducting 3D structural analyses in gel scaffolds.

We herein report the details of our cholangiocyte culture methods and the characteristics of cultured cells and organoids originating from BA patients.

## Materials and methods

### Primary cells

Cholangiocytes were obtained from the contents of gallbladders or from the connective tissues resected from the portal area of livers during the Kasai operation. Informed consent for the research use of resected tissues was obtained from the parents of the patients before the surgery. In our surgical strategy, cholangiography through the gallbladder is usually performed before the Kasai operation to confirm the diagnosis and classification of BA. Before cholangiography, the contents of the gallbladder are aspirated with a fine needle and a syringe and then centrifuged at 2,000–3,000×*g* for 3 min to precipitate the cells. The supernatant is then removed. If the amount of the gallbladder content is small, a few clusters of cells are obtained by washing the needle and syringe used for aspiration in the culture medium.

Connective tissues are surgically resected from the portal area of the liver with the remnants of the disturbed bile ducts in them. Small fragments of resected tissues are then dissected as much as possible using a fine blade and spread in culture medium.

### Primary 2D cell culture and immortalization

Throughout this study, all cells or tissue fragments were cultured at 37 °C with 5% CO_2_. The contents of our conditioned medium are shown in Table 1. A cocktail of Y-27632 (Rock inhibitor), CHIR99021 (GSK-3 inhibitor), and A83-01 (ALK4/5/7 inhibitor) was added to the medium (Table 1) to maintain cell growth (Katsuda et al. 2017), but these compounds were not added to the medium when the cells were used for experiments to avoid the effects of the inhibitors. The medium was replaced every three to four days. After the cells attached to the bottom of dish and started to grow stably, the immortalizing genes SV40T and hTERT were introduced to the cells using a commercially available lentivirus system (Cat# G256, G200; Applied Biological Materials Inc., BC, Canada). Transdux (Cat# LV850A-1; System Bioscience, CA, USA) was added to the culture medium according to the manufacturer’s instructions, and 2–5 MOI (multiplicity of infection) of virus with each gene was introduced to the cells. First, SV40T-Lenti was added to the medium and incubated for one night, and then the medium was changed. After checking that the cells had not been obviously damaged, hTERT-Lenti was added to the medium and cultured overnight. After changing the medium, the culture was continued without adding any selection reagent.

**Table 1:** Components and suppliers used in the conditioned medium

Basic culture medium	Suppliers, catalog #
DMEM/F12 (1:1) w glutamine (2.5 mM)	Nacalai Tesque, Inc. Cat# 08460-95
hEGF 20 ng/ml	Peprotech Cat# Af-100-15
0.1 µM dexamethasone	Nacalai Tesque, Inc. Cat# 11107-64
10 mM nicotinamide (1.22 mg/ml)	Nacalai Tesque, Inc. Cat# 24317-72
Insulin-transferrin-selenium (ITS)	Gibco/Thermo Fisher Scientific Cat# 41400045
MEM nonessential amino acids	Nacalai Tesque, Inc. Cat# 06344-56
Penicillin/Streptomycin	Nacalai Tesque, Inc. Cat# 26253-84

Inhibitors	
10 µM Y-27632	LC Laboratories Cat# Y-5301
3 µM CHIR99021	Nacalai Tesque Inc. Cat# 18764-44
0.5 µM A83-01	Fujifilm Wako Pure Chemical Corporation Cat# 039-24111

For passage, cells were stripped from the dish using Accutase (Cat# 12679-54; Nacalai Tesque, Inc., Kyoto, Japan), incubated for 10 min at 37 °C, and then detached from the bottom by tapping the dish. The cells were washed once with the base medium DMEM/F12 or phosphate-buffered saline (PBS) and then seeded into the new culture medium. Stabilized cells were also cryopreserved for later experiments. Cells were stripped from the dish and washed with PBS once. The cells were then retrieved by centrifuging at 500 × *g* for 2 min, after which the supernatant was removed, and the cells were suspended in the cryopreservation reagent LaboBanker 2 (Cat# BLB-2; TOSC Japan Ltd., Tokyo, Japan) and transferred into cryo tubes. The tubes were placed in a − 80 °C freezer for at least 2 nights and then placed in nitrogen oxide for further preservation.

Thawing was performed according to the manufacturer’s instructions. In brief, the medium was warmed to 37 °C before thawing, and then the tube of frozen cells was thawed in a 37 °C water bath. Cells were transferred into a 10-fold volume of culture medium and centrifuged. The supernatant was removed, and the cells were suspended in new medium and then cultured at 37 °C with 5% CO_2_.

### Immunohistochemistry

Cells were seeded in eight-well glass slides and incubated until they attached to the slide and spread. Attached cells were fixed with 4% paraformaldehyde and incubated for 15 min in room air. The slides were washed and activated in pH 6.0 citrate buffer, heated in a microwave for 10 min with intervals to prevent boiling, and then soaked in methanol with 0.3% H_2_O_2_ for 30 min to inactivate the internal peroxidase. After washing with PBS and incubation with blocking buffer (Blocking One Histo, Cat# 06349-64; Nacalai Tesque, Inc.) for 1 h in room air, the primary antibody (anti-CK19 antibody, 1:200; Cat# ab220193, RRID: AB_2814863; Abcam, Cambridge, UK) was reacted overnight at 4 °C. Slides were then washed, and a secondary antibody (Envision + System-HPR Labeled Polymer Anti-mouse, Cat# K4000; Agilent, CA, USA) reaction was performed for 1 h in room air. After washing, 3,3’Diaminobenzidine (DAB) was used for staining following the manufacturer’s protocol. Cell nuclei were counterstained with hematoxylin for 1 min.

### Immunofluorescence

Cells were cultured in a 24-well dish and fixed with 4% paraformaldehyde for 15 min at room temperature. The plate was then washed, and the cells were permeabilized with PBS with 0.3% Triton X, rocking for 10 min at room temperature. The buffer was replaced with blocking buffer (Blocking One Histo, Cat# 06349-64; Nacalai Tesque, Inc.) and incubated for 1 h at room temperature. The blocking buffer was then removed, and double-primary antibody solution was added to the cells. The antibodies used were 1:200 for CK19 (Cat# ab220193, RRID:AB_2814863; Abcam) and 1:400 for SOX9 (Cat# AB5535, RRID:AB_2239761; Millipore, MA, USA)The plate was placed at 4 °C overnight, and then the samples were washed and incubated with fluorescent secondary antibody solution (both 1:500, Cat# A-11060, RRID:AB_2534107; Cat# A31566, RRID:AB_10374301; Thermo Fisher Scientific, MA, USA) in room air for 1 h. The samples were washed with 0.05% PBS-Tween20 and observed under a fluorescence microscope (BZ9000; Keyence, Osaka, Japan).

### 3D culture

**Fig. 1:**
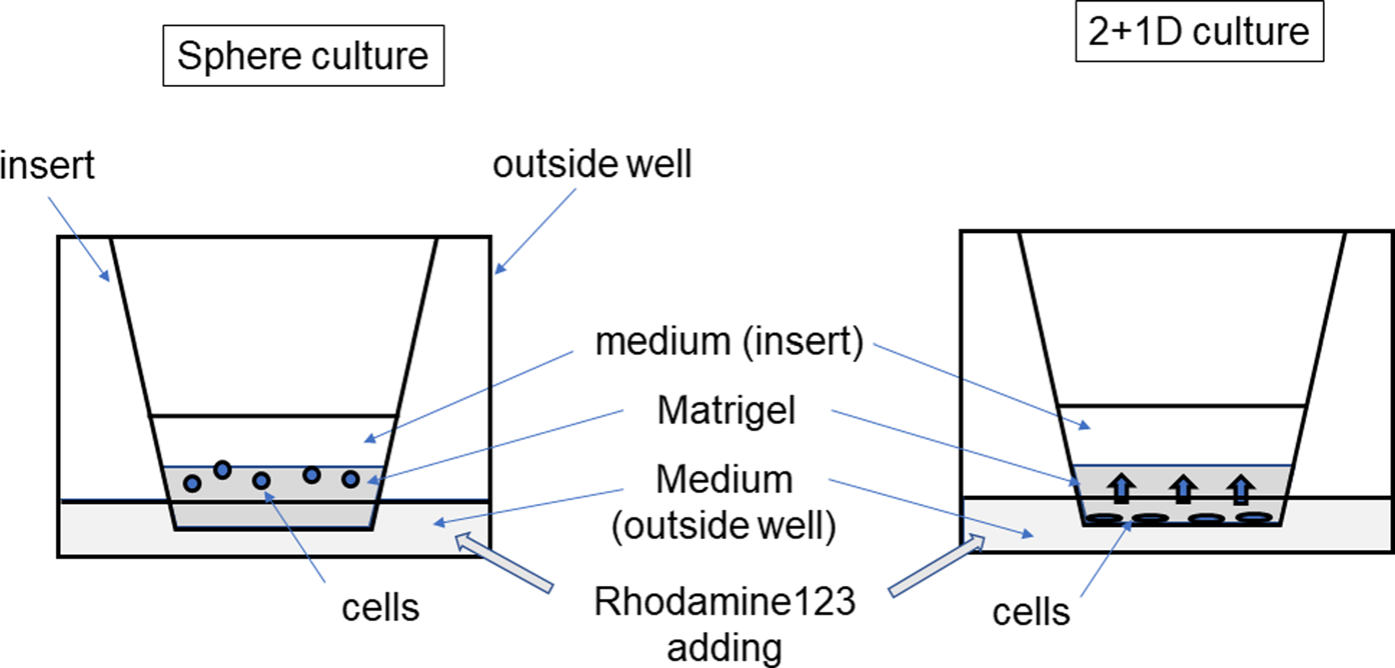
Two 3D culture methods of cholangiocytes. **A** Sphere culture method: cells were seeded on a layer of Matrigel in a 24-well insert. **B** 2D + 1D culture method: cells were first cultured on the membrane of the Transwell insert until the cells covered approximately 50% of the surface, and then Matrigel was placed on top of the cell sheet. Conditioned medium is filled outside and inside the inserts

We tested two 3D culture methods: a Matrigel (Cat# 356234; Corning, NY, USA) and a Transwell system (Cat# 353492; Corning) (Fig. 1). The first method involved spheroid culture, while the second promoted vertical growth from 2D culture (2 + 1D culture).

For the first method (spheroid culture), 100–200 µl of Matrigel was placed in a Falcon Transwell insert for a 24-well culture plate and then incubated at 37 °C. A total of 500 µl of conditioned culture medium was placed in the well to soak the bottom of the insert, and then 200 µl of cell suspension was added to the insert. After cells had formed small spheres in the gel, the culture medium, both in the insert and in the well, was exchanged every three to four days.

For the second method (2 + 1D culture), 2D culture was performed first in the Transwell. A total of 200 µl of cell suspension was placed in the Transwell insert for a 24-well plate with the bottom soaked in 500 µl of conditioned culture medium in the well. Cells were cultured at 37 °C with 5% CO_2_, and the culture medium was replaced every three to four days until the cells grew to cover more than 50% of the membrane. For vertical growth, the medium in the insert was completely removed, and 100 µl of Matrigel was added to the cells and then incubated at 37 °C with the bottom of the insert soaked in medium. After the Matrigel was added, 200 µl of fresh medium was further added to the insert, and the culture was continued until the cells had established 3D structures in the Matrigel.

### The rhodamine-123 uptake in 3D culture cells

Cholangiocytes reportedly take up and excrete rhodamine-123 into bile duct lumens, as has been observed using fluorescence microscopy. Rhodamine-123 (100 µM, Cat# R8004; Sigma-Aldrich, MO, USA) was added to the medium of the 3D culture well in which the 3D culture Transwell had been inserted and then incubated for 30 min at 37 °C until the chemical solution had entered the gel. The well was then washed with PBS three times. The cells were cultured in fresh medium for a few days and observed under a microscope with a 488-nm filter (BZ9000; Keyence). As a negative control, 100 µM verapamil (Cat# 36232-31; Nacalai Tesque, Inc.) was added to the culture medium to inhibit the uptake of rhodamine-123, and the medium was replaced with fresh medium without verapamil after overnight incubation.

## Results

### Primary 2D culture of cells from bile tracts of BA patients

**Fig. 2:**
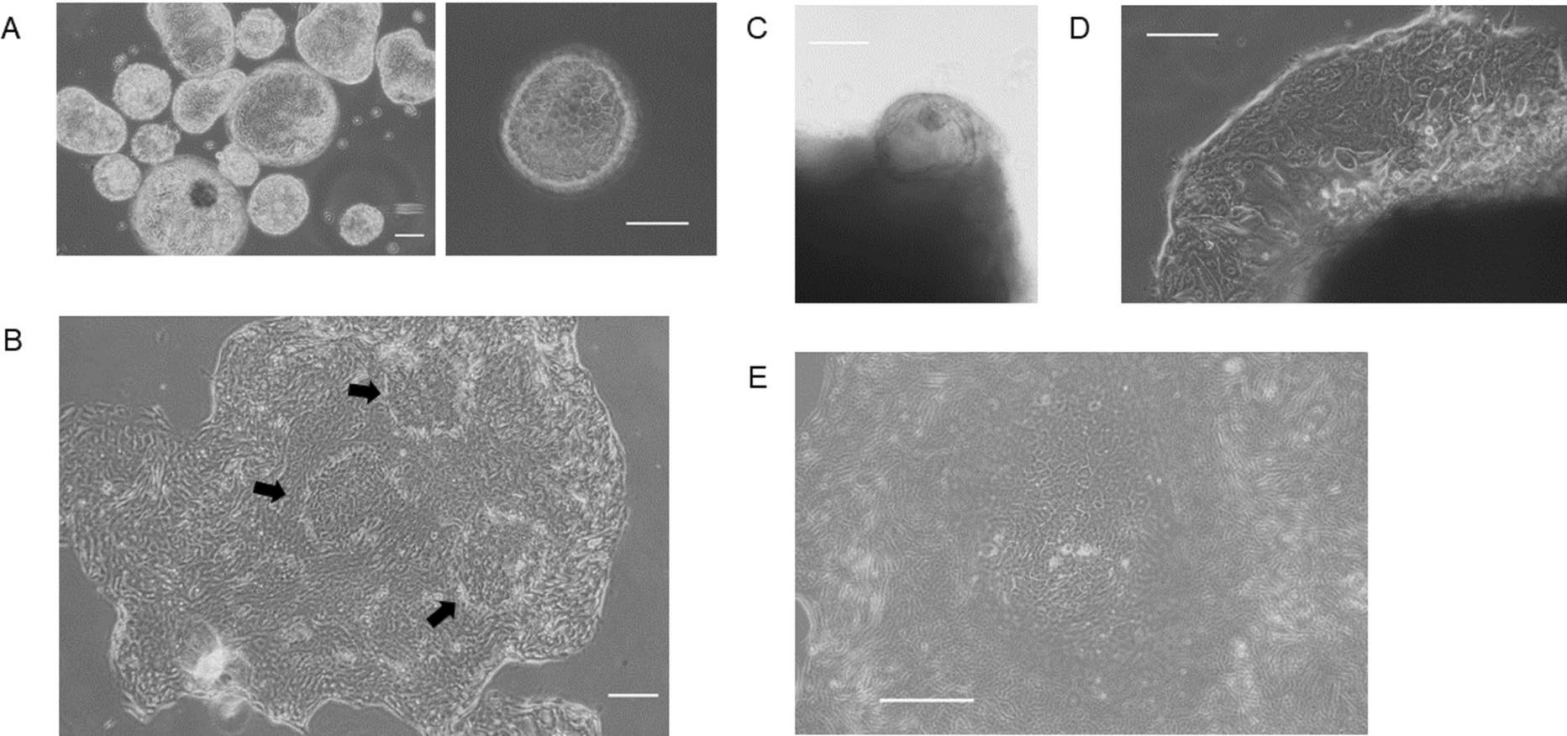
Primary cell culture and 2D growth. **A** Cells obtained from gallbladders formed spheres. **B** Spheres attached to the bottom of the culture plate. Clusters of small cells (arrows) were found among the spread cells. **C** Masses formed from the tissue fragment of the liver portal area. Once attached to the plate, the cells spread around the tissue (**D**). **E** Amplified view of a small cell cluster. Scale bar = 100 μm

The cells obtained from gallbladders formed spheres in the culture medium after a few days of incubation (Fig. 2A). Cells grew as spheres to some extent and then tended to attach to the bottom of the dish and spread around (Fig. 2B).

Regarding the culture of tissue fragments from the portal area, round masses were formed from the tissues after a few days of incubation in the conditioned medium (Fig. 2C). Once the tissues had attached to the bottom of the dish, nascent cells grew around the masses and spread (Fig. 2D). At first, the cells were smaller than the cells from gallbladders and then grew larger while spreading. It had been previously assumed that the surgically resected tissues contained several types of cells, particularly fibroblasts, which were expected to comprise most of the components. However, amplified cells showed almost a single form, and fibroblastic cells did not grow in our conditioned medium at all. Furthermore, the cells were sorted during repeated subcultures, and after a few passages, they showed an even form.

Amplified cells from both origins were relatively broad, cytoplasm-rich shapes with blunt angles. However, there were clusters of small cells among the spread cells (Fig. 2B, allows, 2E). These cells appeared to be rather immature amplifying cells that eventually differentiated into larger cytoplasm-rich mature cells while spreading around. After immortalization by SV40T and hTERT inductions, the cells continued to grow with no morphological changes. In addition, these cells were well tolerated and grew after passaging or thawing.

### Immunological staining for confirmation of cholangiocytes

**Fig. 3:**
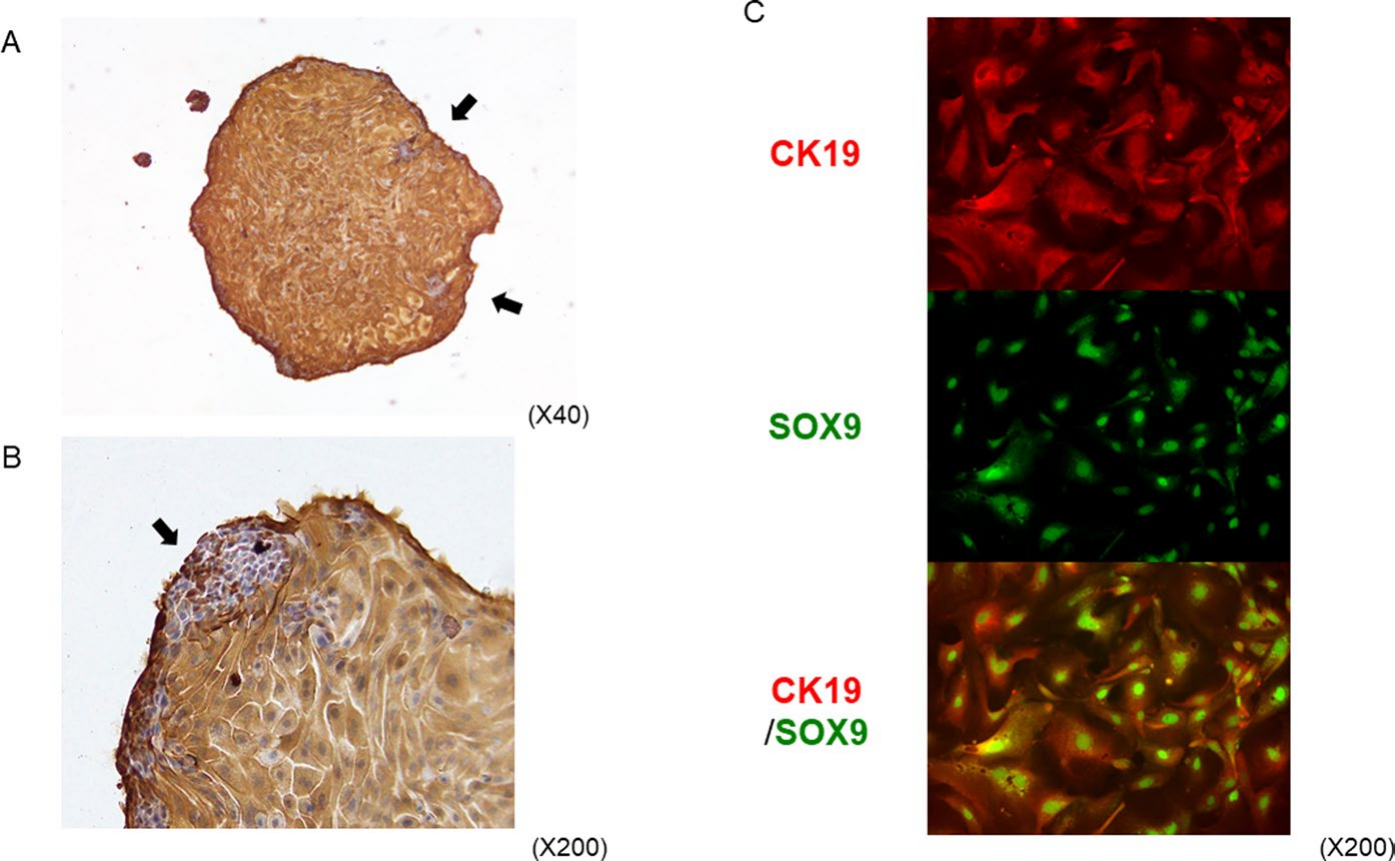
Immunostaining of cells. **A** Immunohistochemistry of cells with CK19. Cells were mostly positive for CK19. However, focal clusters of small cells showed weak staining (**B**, arrow). **C** Double immunofluorescence staining for CK19 (red) and SOX9 (green). Cells were positive for both antibodies. (Color figure online)

Immunohistochemistry and double-staining immunofluorescence were performed to confirm the cells as cholangiocytes. Cells that were attached and fixed to glass slides showed positive staining with anti-CK19 antibody (Fig. 3A, B). The staining was strong in the broadly spread cells, but the clusters of small cells showed weaker staining than the surrounding cells (Fig. 3B).

In addition, double-staining immunofluorescence with anti-CK19 and anti-SOX9 antibodies showed positive staining for both in the cells (CK19 in the cytoplasm and SOX9 in the nucleus; Fig. 3C).

These immunological staining patterns were similar in both cells from gallbladders and from tissues of the portal area.

### 3D organoid culture and the rhodamine-123 uptake

As described above, we developed two different organoid cultures systems: one forming spheroids and one grown from a 2D sheet of cells in the vertical dimension (2 + 1D culture).

**Fig. 4:**
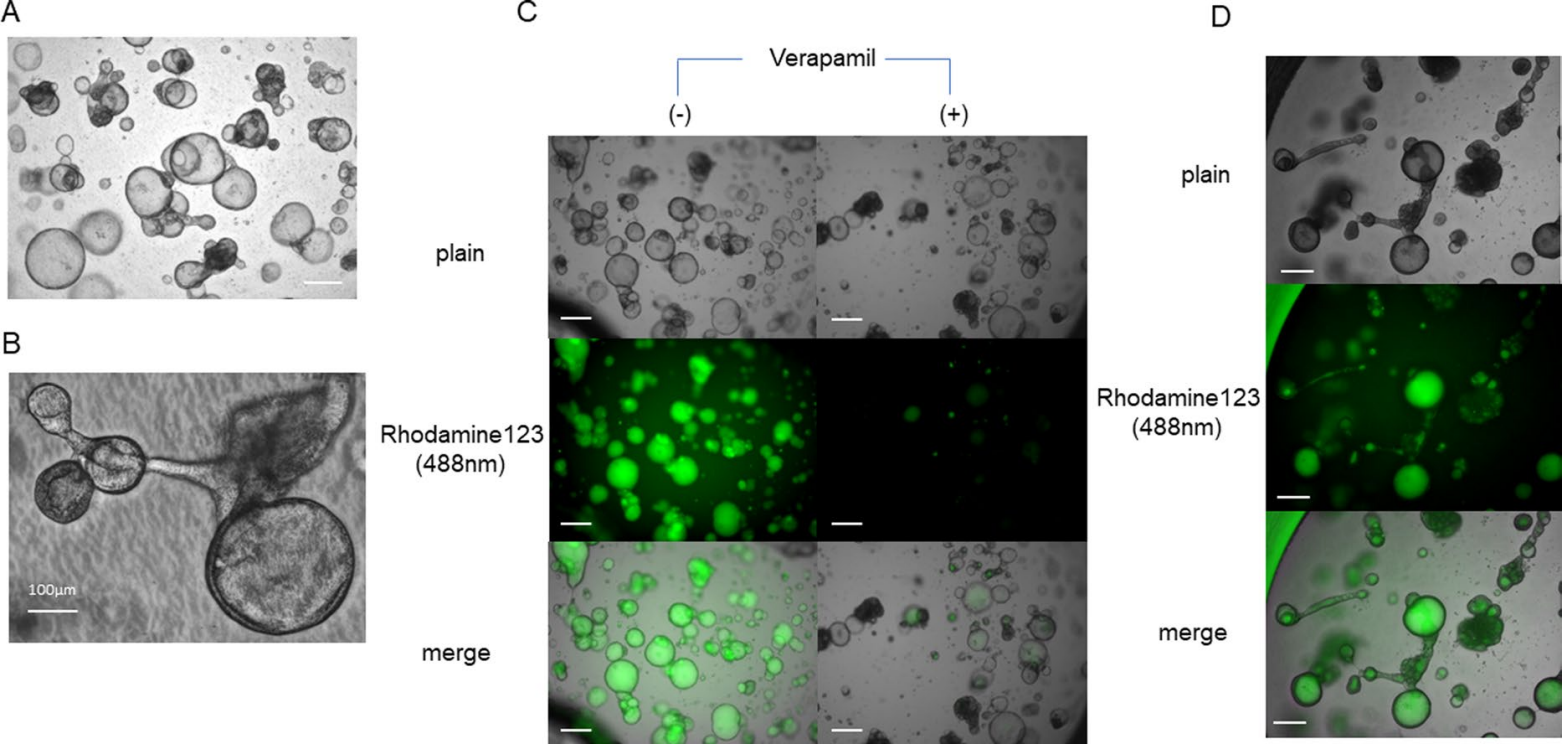
Sphere culture of cholangiocytes in Matrigel. **A**, **B** Plain view of sphere cultures. Spheres form cysts and extend ducts to connect with each other (**B**). **C** Rhodamine-123 was taken into the spheres (green), while the signal was weak with verapamil. (**D**) Rhodamine-123 was also found in the ductal structures (arrows). Scale bar (**A**, **C**, **D**) = 400 μm. (Color figure online)

Cells seeded on Matrigel in the Transwell inserts of 24-well plates formed spheres within a week (Fig. 4A). The cells from the portal area of the liver showed more balloon-like spheres than spheres from gallbladders. The spheres grew to some extent, maintaining their round shape in the gel, and then tended to connect with each other, extending narrow duct-like structures beyond their boundaries (Fig. 4B, D). Rhodamine-123 was taken up into the spheres a few days after induction, and this uptake could be observed under a microscope for several days up to a few weeks. Spheres from portal area cells showed the uptake of fluorescence in the whole spheres (Fig. 4C, D), while the spheres from gallbladder cells showed mosaiced fluorescence inside the spheres, indicating that the sphere was filled with cells. The rhodamine-123 fluorescent signal was reduced by incubation with verapamil in the medium (Fig. 4C). Verapamil might influence the growth of spheres, as the shape of the spheres seemed irregular in the long exposure to the chemical, so the medium was replaced after overnight incubation. However, even over 24 h after the medium change, the fluorescence remained weaker in spheres incubated with verapamil than in those without it. Fluorescence was also observed in the narrow duct-like structures connecting spheres or extended in the Matrigel outside the spheres (Fig. 4D).

**Fig. 5:**
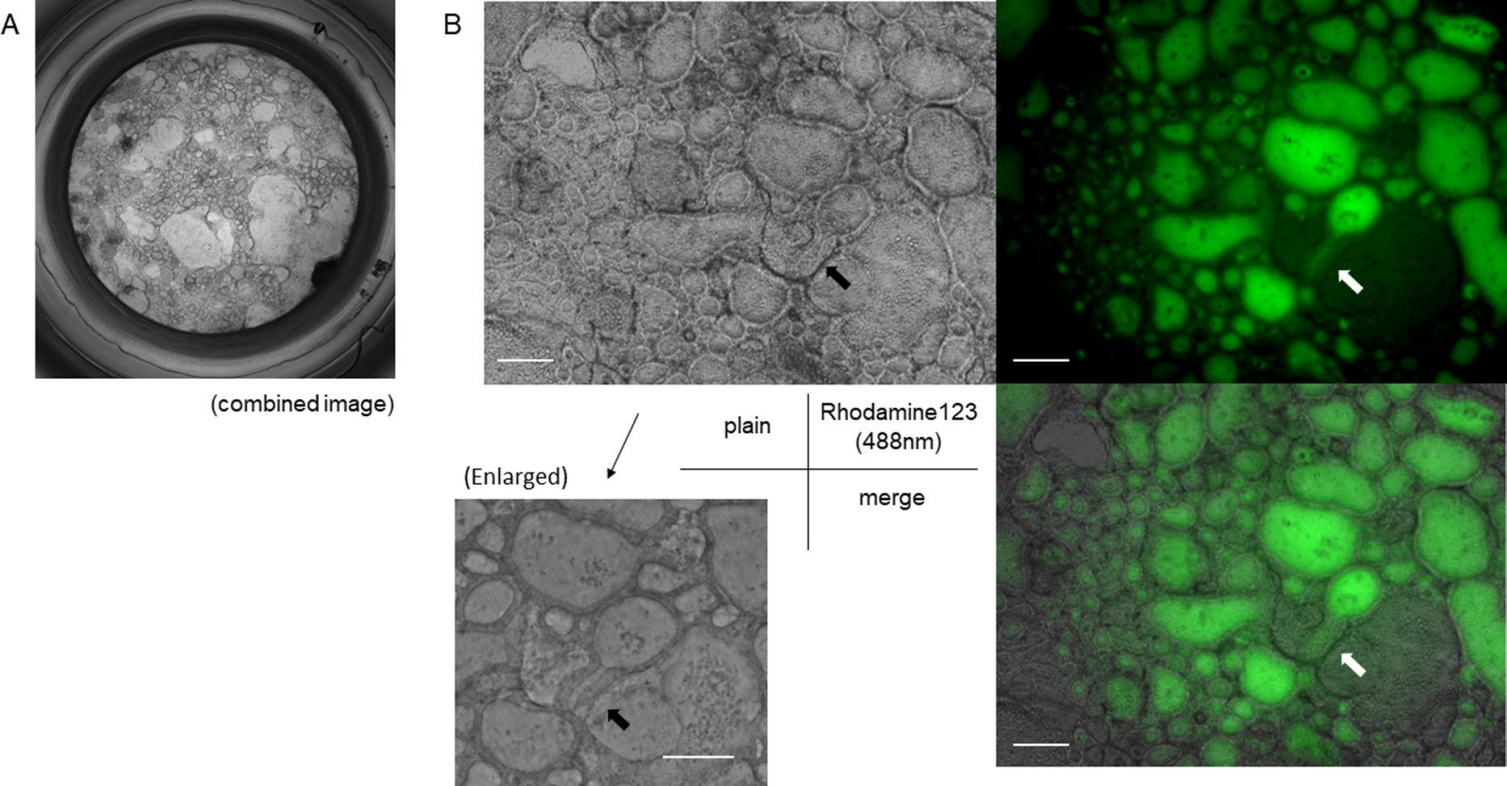
The 2D + 1D organoid culture of cholangiocytes in Matrigel. **A** A plain view of the whole bottom of the insert. The image is a combined view of pieces of ×40 images. The organoids formed a honeycomb structure with various-sized hollows on the bottom of the insert of the Transwell. **B** Rhodamine-123 accumulated in the lumen of the hollow structures (green). Rhodamine-123 was also found in the ductal structures (arrows). Scale bar = 400 μm. (Color figure online)

In contrast, the 2 + 1D culture showed a honeycomb-like appearance at the bottom of the Transwell while growing in the Matrigel (Fig. 5A), and lines of epithelium-like cell sequences surrounded the hollow lumens (Fig. 5B). After incubation with rhodamine-123, the organoid showed aggregated fluorescence inside the hollows (Fig. 5B). The surrounding linings of cells were also positive for fluorescence, but the signal was stronger inside the lumens. There were some narrow ductal shapes that extended from the hollows, seeming to connect one hollow to another. Fluorescence was also found in the lumen of the ducts.

## Discussion

BA is the most frequent cause of liver cirrhosis and transplantation in children. Cholestasis due to primary bile duct obstruction can persist even after primary surgery, leading to liver failure (Kasahara et al. 2017; Rodijk et al. 2020; Ziogas et al. 2021).

We developed our cholangiocyte culture system to analyze the dynamics of cholangiocytes in vitro with the goal of investigating not only the cause of bile duct damage but also the process of bile duct regeneration at the portal area of the liver after surgery (e.g. determining which factors promote bile duct synthesis). There have been several reports of cholangiocyte cultures from animals (Sampaziotis et al. 2017; Chen et al. 2023) and humans (Amarachintha et al. 2022; Tysoe et al. 2019; Costantini et al. 2019; Karjoo et al. 2014). Most involved primary cultures from liver tissues and were related to regeneration research. In the present study, we specifically developed stable cell culture systems to analyze epithelial cells in extrahepatic bile ducts, which are the focus of pathogenesis in BA patients.

Difficulties in the stable culture of normal human cholangiocytes include the following: (A) obtaining and selecting cells, (B) maintaining cell growth, and (C) escaping senescence and cell death. In our method, (A) obtaining and selecting cholangiocytes were relatively easy, as we were able to obtain cells during BA surgery, and fibroblasts or other cells usually did not grow in our conditioned culture medium. Once cells were obtained from the gallbladder or the portal area of the liver of BA patients, we simply cultured them in our conditioned medium, and cholangiocytes were automatically selected eventually. The morphological features of cholangiocytes were relatively easy to identify, and the cells were confirmed by immunostaining with the cholangiocyte marker CK19. Regarding (B), maintaining cell growth is usually difficult for mature cholangiocytes. We therefore added a cocktail of inhibitors Y-27632 (Rock inhibitor), CHIR99021 (GSK-3 inhibitor), and A83-01 (ALK4/5/7 inhibitor), referring to a liver regeneration research report (Katsuda et al. 2017) and these inhibitors maintained the immaturity and amplifying ability of the cultured cells. Without these inhibitors, our cells did not grow and eventually died after approximately one week, even with the induction of SV40T and hTERT. Given the effects of these compounds, we excluded them from the culture medium when the cells had been planned to be used for experiments. Regarding (C), escape from senescence and cell death was achieved by introducing the immortalizing genes SV40T and hTERT. After the induction of these genes, cells did not show morphological changes and grew stably.

The composition of our conditioned culture medium was referenced from previously reported human cholangiocyte cultures (Kubota and Reid 2000; Karjoo and Wells 2014; Sampaziotis et al. 2017; Tysoe et al. 2019; Costantini et al. 2019) mainly based on Kubota’s culture medium, which was reported to be suitable for cholangiocyte culture (Kubota and Reid 2000; Costantini et al. 2019). We arranged the composition by selecting the most commonly used components among them; thus, the components were relatively simple compared to other reported components. Although our culture medium contains some inhibitors that maintain the immaturity of cells, most of the cells actually show rather differentiated features and develop ductal structures in organoids. The organoid also took up rhodamine-123, indicating that the cells possess cholangiocyte functions (Gigliozzi et al. 2000; Tanimizu et al. 2007; Amarachintha et al. 2022; Chen et al. 2023). In the 2D culture, amplifying cells also developed clusters of small cells that were not stained with CK19 immunohistochemically. These small cells appeared to be immature cells that formed clusters at the center of cell amplification, while the differentiating cells spread around. The mixture of immature cell amplification and cell differentiation seems to be well balanced in our culture system.

We intended to use these cells or organoids in our experiments, such as for examining the effects of cytokines or chemicals added to the medium or observing the growth patterns of cells forming structures. We developed two different 3D culture systems: sphere culture and 2D + 1D culture. The spheroids showed the formation of ductal structures well, and the 2 + 1D culture seemed to show the insides of 3D structures. The sphere culture extended narrow ducts from one sphere to another, such as regenerating bile ducts in which cells take up substances and excrete them into the lumen, trying to transfer them as the bile ducts do in vivo. The 2D + 1D culture appeared similar to the tissue sections observed under microscopes, engaging in living cell activities, such as taking up and excreting rhodamine-123.

We used an in vitro system to examine the reaction of cholangiocytes by cytokines or chemicals, excluding the effects of other cells, such as immune cells or fibroblasts. It is simpler to induce the effects of substances in cultured cells and observe the morphological or growth changes or to examine the RNA or protein expression in those cells by harvesting them from dishes than examining in vivo model. Since these cells tolerate cryopreservation and thawing, it is also possible to repeat experiments after cells have stabilized. Even though the environment is different from that in vivo, we can easily examine our hypothesis with this system. It is also easy to observe the construction of bile duct extension in our 3D culture system, even without the scaffold of other types of cells.

We reported methods of stable 2D culture and 3D organoid culture of cholangiocytes from BA patients. Our methods are relatively simple to perform and easily available, and the cultured cells are functional as cholangiocytes. We hope that in vitro systems such as ours will help clarify the etiology and development of therapy and thus improve the prognosis of BA patients.
